# Ten Cases Illustrating the Value of Geranium Maculatum

**Published:** 1889-10

**Authors:** John V. Shoemaker

**Affiliations:** Philadelphia, Pennsylvania


VOL. VI. OCTOBER, 1889. NO. 8.

### Original Communications.

### TEN CASES ILLUSTRATING THE VALUE OF GERANIUM MACULATUM.

BY JOHN V. SHOEMAKER, A. M., M. D.,
PHILADELPHIA, PENNSYLVANIA.

About two years ago I called attention to the value of Geranium Maculatum* in incipient phthisis and hæmoptysis and as a general astringent and hæmastatic.

INCIPIENT PHTHISIS.—The following abstracts from my note-book since then will show that it is worthy of a high place as a remedial agent.

CASE I.—D. M., aged 24, came under my care in November, 1887. He had been suffering from a harassing cough and copious night sweats for four weeks. His appetite was poor, his sleep restless and he had lost twelve pounds in weight. His father and mother had both died from consumption, and he was

* Geranium Maculatum, a paper read before the American Medical Association on June 7th, 1887.

naturally very much alarmed lest he would be carried off by the same dread disease. Auscultation revealed marked dullness over the left apex, and prolonged expiration on both sides. His pulse was 110 and weak, he denied ever being feverish but stated that his cheeks flushed up in the afternoon.

As I had seen several similar cases in which the pulmonary dullness was due to malaria and disappeared under quinine, and as the weather at the time he was taken sick was dull and heavy, I gave him full doses of quinine for a week but without any good results. I then ordered him quinine in the morning, a cough mixture through the day, and belladonna and aromatic sulphuric acid at night, but without securing any improvement. The quinine was then stopped, and blisters applied to the left apex, but the patient grew worse. Finally, and as a last resort, I prescribed two ounces of the fluid extract of Geranium and with instructions to take a teaspoonful of it in water before meals and at bedtime. To my surprise and delight he commenced to improve at once, the cough lessened in severity, the night sweats disappeared, appetite improved, pulse gradually went down to 84, and after three weeks further employment of geranium he considered himself so well that the treatment was discontinued.

About two months ago he stopped in to tell me of his marriage, and that his wife had just given birth to a son. He further reports an increase in weight of ten pound, and an apparent restoration to health.

CASE 2.—Incipient phthisis, M. J., aged 28, came to me in January, 1888. He was a weaver by trade, and worked in a room the air of which was full of fine dust. He had been troubled for three weeks with a violent paroxysmal cough and night sweats. Auscultation disclosed clicking inspiration, and prolonged expiration over the upper lobe of the left lung. He had been losing in weight and appetite. His pulse was 116 and weak. I placed him on drachm doses of the fluid extract of Geranium, to be taken well diluted every four hours. When he returned ten days later his pulse had fallen to 100, the cough had lessened and the night sweats had ceased. After another week of Geranium his pulse went down to 92, and there was further improvement in all his symptoms.

I did not see the patient again for a month when he came in to say, that as he had to work over time, he could not come to my office, but that the medicine had been renewed four times and he thought that he was nearly cured by it. His pulse was then 84. The physical signs had disappeared and he only coughed about four times a day. I advised him to continue medical treatment for a while longer. He did so, and I had the pleasure of discharging him two months later, ten pounds heavier, feeling well.

CASE 3.—Phthisis, Miss J., aged 19, complained of a slight chill on February 12th, 1888, and went to bed. She had previously been in apparently the best of health, although always of an extremely pallid complexion. I saw her on the following day, and did not regard her as being seriously ill. Before the week was out, however, I deemed it my duty to tell her parents that I believed she was going into quick consumption and that she might not live three months. I based this opinion upon the following symptoms: viz, a persistent temperature of 102, unaffected by either quinine, salicylic acid or aconite, a weak and rapid pulse ranging from 120 to 130, and an apparent solidification of the upper posterior portion of the middle lobe of the right lung, accompanied by increased rapidity of breathing, but not attended by any pain, discomfort, cough or expectoration. Of course I endeavored to soften the blow to her affectionate parents, by saying that there was a probability of my diagnosis being erroneous, and advised them to give me the privilege of bringing in a consultant. The consultant physician whom we agreed upon came and saw the patient, and decided that while it was well to regard the case as serious, still he did not think it was as grave as I dreaded, and that it was more analogous to pneumonia than to phthisis. I adhered to my opinion, but stated that I would adopt any method of treatment that would cure or relieve the patient. A line of medication was agreed upon and faithfully observed for three weeks, but so far from any improvement being evident, the patient's condition grew worse every day. The family then concluded to recommit the patient to my unaided care. During the interim, ammonia, in its various forms, cresote, iodine, quinine, salicylic acid and other anti-pneumonia remedies,with stimulants,had been tried and abandoned as useless. I stated to the parents that I had no hope of being able to effect a cure but that I would try to prolong her life as long as possible. At this time she was not able to sit up on the bed and was suffering from constant and harassing cough, complete anorexia and profuse night sweats. In prescribing, I directed her to take a teaspoonful of the following formula every three hours:

℞—

Ol Menth. Pip.................................mxx

Ext. Geranii Fl.................................℥iss

Vini Portensae................................. ℥ i

Mix.

She began to improve on this prescription, and in less than three weeks was able to get out of bed and sit up. The following week she was strong enough to walk down stairs. I was glad to note the improvement under treatment, even if it did point to a failure in my diagnosis or prognosis. The family frequently joked with me on the subject, and my reply was "I hope the improvement will continue, if so I will be able to make public a certain remedy for consumption."

The improvement did not continue, however, other portions of the lung substance became invaded, increasing weakness compelled her to go to bed again, and in less than five months from the date of her first illness she died.

CASE 4.—HÆMOPTYSIS—J. D., aged 25, had three attacks of hæmoptysis in 1885, or rather one attack lasting a week, during which three hemorrhages occurred. I treated him at that time with witch-hazel and port wine, given alternately with sulphate of magnesia and dilute sulphuric acid. He was a month convalescing. In March, 1888, he had another attack and again sent for me. His general condition was worse than in 1885. His pulse was quick and weak, and the probabilities were in favor of a recurrent hemorrhage. I ordered him to take two drachms of the fluid extract of Geranium at once, and then to continue with one drachm regardless of whether vomiting or hemorrhages supervened. On my return next day the patient was feeling comparatively well and strong. His sputa was tinged with blood but no large quantity had appeared. He had comparatively little cough and his pulse was only 90 and fairly strong. He kept up the Geranium for two weeks, when he went to work and has enjoyed comparatively good health to the present time.

CASE 5.—GASTRIC ULCER.—Miss A., aged 21, had been dyspeptic for two years and for several months had been troubled with a sharp pain in the back after eating. She had taken several patent medicines without benefit. In June, 1888, she fainted on the street. On reviving she was taken home. That evening, just as she was going to sit at the supper table, she vomited some thick black material and in a few minutes afterwards brought up a small amount of pure blood. On seeing her about two hours later, I informed her family that it was a case of gastric ulcer, and ordered a teaspoonful of the fluid extract of Geranium every two hours that night, and every three hours the following day. The patient had no further relapse, and after a month's use of Geranium, and perfect rest, returned to her work as a bookkeeper and has been ever since in good health.

CASE 6.—ANAL FISSURE.—J. F., aged 26, came to me complaining of constant itching at the margin of the anus, complicated by excruciating pain whenever his bowels moved. An examination revealed a fissure three-eighths of an inch long and about two lines deep, at the line of junction of the skin and mucous membrane. I ordered him four ounces of a twenty-five per cent. solution of the fluid extract of Geranium and told him to apply a rag dipped in it to the anus night and morning. These applications produced a complete cure.

CASE 7.—URETHRAL HEMORRHAGE ARRESTED BY GERANIUM.—J. F., aged 28, had been suffering from gleet accompanied by pain upon erection for two years. Last November, after indulging freely in beer, an unusual firm erection occurred, during which he felt something break in the urethra. This was succeeded by a sharp pain and a sensation as if there was something moist escaping. On making an examination he was surprised to find a tiny stream of blood oozing from the urethra. He applied pressure to the penis, but without effect. He then ran for assistance and fainted. When I arrived at his room a few minutes afterwards, I found that he had lost considerable blood and that the hemorrhage was still unchecked. I gave him an injection composed of one drachm of fluid extract of Geranium and four drachms of water and kept it in the urethra a few moments; a fresh injection of the same quantity and strength, retaining it also, was again repeated. This simple procedure not only checked the hemorrhage, but had the unexpected result of curing the gleet, which for a long time had defied all treatment.

CASE 8.—METRORRHAGIA.—Mrs. H., aged 25 years, had a miscarriage at two months, which was followed by an obstinate metrorrhagia. The os was dilated and the interior of the uterus carefully examined, but no retained membrane or other offending material could be discovered. The usual methods of treatment were carried out for four weeks, but without any improvement. Finally an intra uterine injection of half an ounce of Geranium and half a pint of water was resorted to as an experiment. It gave such good results that it was repeated twice a day for two days, once daily for three days, and after several succeeding injections the patient was discharged cured.

CASE 9.—BUCCAL ULCERATION.—Mary F., aged three months, refused to nurse for three days, during which time she cried almost constantly. There was no abnormality of pulse or temperature, no pain on pressure of the groins or abdomen, but examination of the roof of the mouth revealed an oval ulceration about half an inch long and quite deep. The healthy appearance of both parents and child dispelled any suspicion of syphilis that might have been entertained. Four applications of a twenty per cent. solution of Geranium eased the child and permitted it to nurse again, and its daily repetition for a week effected a complete healing of the sore.

CASE 10.—BUCCAL ULCERATION.—A young man, aged 22, came in my office saying that he had "cut a tooth" two days before, and that it had made his mouth so sore that he could not eat, and he wanted to get something to relieve the pain. On looking at the inner surface of his mouth, I was unable to find any evidence of the appearance of a new tooth, but I saw that he had an extensive ulceration of the lower gum and the adjacent buccal membrane.

I ordered him to paint the affected surface every three hours with a twenty-five per cent. solution of the fluid extract of Geranium, and abstain from hot, acid or otherwise irritating articles of food. He returned in four days cured.

*1519 Walnut Street.*

